# Voltammetric Detection of Chlorophenols in Brewing Water and Beer Using Different Carbonaceous Composite Electrodes

**DOI:** 10.17113/ftb.63.03.25.8814

**Published:** 2025-09-26

**Authors:** Sali Muriqi, Libor Červenka, Milan Sýs

**Affiliations:** Department of Analytical Chemistry, Faculty of Chemical Technology, University of Pardubice, Studentská 573, 532 10 Pardubice, Czech Republic

**Keywords:** beer, brewing water, chlorophenols, electrochemical detection, liquid chromatography, food quality

## Abstract

**Research background:**

Nowadays, there is still no portable electroanalytical device suitable for the monitoring concentration of chlorophenols in technologically used water, especially in the brewing industry. This problem could be solved by developing an electroanalytical screening method based on chlorophenol anodic oxidation.

**Experimental approach:**

The electrochemical behaviour of the target chlorophenols was investigated to find the optimum working conditions for their selective electrochemical detection in beer.

**Results and conclusions:**

Electrochemical oxidation pathways were proposed for each investigated chlorophenol. The sum of all chlorophenols present in the brewing water, expressed as the concentration equivalent of 2,6-dichlorophenol, can be determined electrochemically, so that in future real-time monitoring of chlorophenols in the individual stages of the beer production process will be possible. Moreover, the cathodic reduction of their oxidation products proved to be a suitable electroanalytical tool for the selective detection of their presence in beer.

**Novelty and scientific contribution:**

The research shows that an electroanalytical approach could be useful in the control of beer biotechnology to prevent sensory changes caused by the chlorophenols formed.

## INTRODUCTION

Beer contains naturally occurring phenolic compounds, especially polyphenols. They are derived directly from hops and malt (*1*). Chlorophenols are formed by chlorine and chloramine in the brewing water, which bind to the polyphenol metabolites produced by the yeast during fermentation (*2*). Contamination by wild yeasts and insufficient removal of chlorine-based cleaning agents can be considered further sources of chlorophenols in beer (*3*, *4*). The yeast binds these phenols to the chlorine compounds to protect them, but the result is a beer with a rather undesirable taste. This is the main reason why chlorophenols are responsible for specific off-flavour in beer, described as clove, medicinal, smoky or "band-aid" (*5*). For example, 2,6-dichlorophenol (2,6-DCP) is considered a defect in any beer if you can smell the typical flavour of mouthwash. The cause of this defect is usually poorly washed packaging or tanks after sanitation. Additionally, it has been experimentally proven that 2,6-DCP can be formed by the gradual chlorination of 2-chlorophenol (2-CP) even to 2,4,6-trichlorophenol (2,4,6-TCP) during the usual water chlorination with chloramine (*6*). Since these chlorophenols can be expected in brewing water and especially in top-fermented wheat beer (*7*, *8*), they became the subject of this electrochemical study. Basic technological guidelines for minimising the formation of sensory active chlorophenols in beer are listed below.

### Prevention of the occurrence of chlorophenols in beer

Chlorophenols can be minimised by the following measures: (*i*) using yeast cultures that produce fewer phenols, (*ii*) pure yeast cultures must be used to avoid contamination by wild yeasts, (*iii*) tap water should be filtered, boiled or at least allowed to stand to remove free chlorine present in concentrations of 0.05 to 0.25 mg/L, (*iv*) if chlorine-based cleaning products are used, all equipment must be thoroughly washed, or such products should be avoided, (*v*) malt should not be ground very finely to prevent increased release of phenolic substances, (*vi*) sweetening water must be kept at pH=6 and below 76 °C, and (*vii*) fermented alcoholic drinks (FADS) such as non-alcoholic (NA) and low-alcohol (LO-ABV) beer below *φ*(EtOH)~0.82 % should not be considered.

Chlorophenols in beer can be observable at very low concentrations. These compounds have intense aroma and flavour even at small quantities. Unfortunately, the specific threshold concentration is not explicitly stated, but their presence is monitored in brewing. The threshold concentration of chlorophenols in drinking water varies according to different standards and recommendations. For example, the US Environmental Protection Agency (EPA) requires 0.2 mg/L with a maximum of 4.0 mg/L, while the World Health Organization (WHO) allows up to 5 mg/L (*9*). In the Czech Republic, Decree No. 252/2004 Coll. sets hygiene requirements for drinking water, namely 0.1 μg/L 2-CP (*10*).

Due to their low concentration, numerous voltammetric methods based on anodic oxidation using predominantly modified sensors have already been developed. Table 1 contains only a few examples (*11*-*21*) which show that a selective voltammetric method has not yet been developed for the single or simultaneous determination of chlorophenols. The main reason for the impossibility of their selective determination could be their similar electrochemical properties, which result in relatively broad anodic peaks. Therefore, it seems that only the sum of all chlorophenols present in brewing water and beer can be determined voltammetrically.

**Table 1:** Overview of several voltammetric methods designed for determination of chlorophenols

Sensor	Analyte	Sample	Interference	Reference
GCE/HP-β-CD-GNR	2-CP	Water	4-CP, 2,4,6-TCP	(*11*)
GCE/MWCNTs-DCP	2-CP	Water	PhOH, 4-CP, 2,4-DCP, 2,4,6-TCP	(*12*)
GCE/AuNPs@cMWCNT	4-CP	Water	PhOH	(*13*)
GPSZnH	4-CP	Water	Not investigated towards other chlorophenols	(*14*)
CPE/Lap	2,4-DCP	Water	2-CP, 4-CP	(*15*)
GCE/MIPs/PRGO	2,4-DCP	Water	Not investigated towards other chlorophenols	(*16*)
GCE/Dy_2_O_3_-Co_3_O_4_@CeO_2_	2,6-DCP	Water	Not investigated towards other chlorophenols	(*17*)
GCE/AlSiFe	2,6-DCP	Water	2-CP, 4-CP, 2,4-DCP, 2,4,6-TCP	(*18*)
BDDE	2,4,6-TCP	Beverages	Not investigated towards other chlorophenols	(*19*)
GCE/Nafion/CuO	2,4,6-TCP	Water	Not investigated towards other chlorophenols	(*20*)
ITO/SAM/AuNPs/HS-β-CD	2,4,6-TCP	Water	2-CP, 4-CP, 2,4-DCP	(*21*)

AuNPs@cMWCNT=gold nanoparticle/carboxyl functionalized multi-walled carbon nanotube, BDDE=boron-doped diamond electrode, CPE/Lap=laponite-modified carbon paste electrode, GCE=glassy carbon electrode, GPSZnH=graphite paste modified with metallosilsesquioxane, HP-β-CD-GNR=graphene nanoribbon functionalized with 2-hydroxypropyl-β-cyclodextrin, ITO/SAM/AuNPs/HS-β-CD=HS-β-cyclodextrin/gold nanoparticle composite-modified indium tin oxide electrode, MIPs/PRGO=molecularly imprinted polymer with *Carica papaya*-reduced graphene oxide, MWCNTs-DCP=multi-wall carbon nanotube-dicetyl phosphate film. CP, DCP and TCP=chlorophenol, di- and trichlorophenol, respectively

As shown in Table 1, various modified electrodes, mainly glassy carbon electrodes (GCE), have been used in the development of voltammetric methods for the determination of chlorophenols in tap water. Two recently published scientific papers suggest that composite carbon materials could also find their application in chlorophenol electrosensing. These reports from 2023 include graphitic carbon nitride nanosheets with Fe_3_O_4_ nanosphere composite-modified screen-printed carbon electrodes for real-time detection of 2,4,6-trichlorophenol in environmental samples (*22*) and carbon paste electrode (CPE) modified with cellulose nanofibers containing Fe_3_O_4_ for monitoring 4-chlorophenol as a water pollutant (*23*). Both electroanalytical methods were also based on direct anodic oxidation using pulse voltammetric techniques and were not sufficiently selective to distinguish individual chlorophenols. A fundamental lack of knowledge about the electrochemical behaviour of chlorophenols is the key reason why their subsequent electrode reactions were not favoured.

In this study, the electrochemical behaviour of different chlorophenols at composite carbonaceous electrodes was therefore investigated. A conventional CPE (*24*), CPE prepared from highly conductive spectroscopic graphite powder (GPE), CPE modified with *w*=5 % multi-walled carbon nanotubes (CPE/MWCNTs) and glassy carbon paste electrode (GCPE) were used. Phenol (PhOH) and three chlorophenols with various numbers and positions of chlorine atoms in the benzene rings, namely 2-chlorophenol (2-CP), 2,6-dichlorophenol (2,6-DCP) and 2,4,6-trichlorophenol (2,4,6-TCP), were studied in different aqueous media using cyclic voltammetry at different scan rates over the potential range of −0.6 to +1.6 V *vs.* silver/silver chloride reference electrode. A square wave anodic voltammetric method was proposed to find whether it is possible to adjust the working conditions so that each investigated chlorophenol gives comparable current yields of the electrode reactions.

## MATERIALS AND METHODS

### Chemicals and reagents

Phenol, 2-chlorophenol, 2,6-dichlorophenol and 2,4,6-trichlorophenol were obtained from Merck KGaA (Darmstadt, Germany). Their stock solutions with a concentration of 0.01 mol/L were prepared in 10-mL volumetric flasks. Due to the low solubility of 2,6-dichlorophenol and 2,4,6-trichlorophenol in pure water, all standards had to be dissolved in absolute ethanol (EtOH) of ≥99.5 % purity. Glacial acetic acid, sodium acetate, disodium hydrogen phosphate, sodium dihydrogen phosphate dihydrate, ammonium chloride and *w*=24 % ammonia solution were purchased from Lach-Ner, s.r.o. (Neratovice, Czech Republic) and used for the preparation of 0.1 mol/L acetate buffer (AcB) with a pH=4.5, sodium phosphate buffer (SPB) with a pH=7.0 and ammonium buffer (AmB) with a pH=10. An ultrapure water with resistivity lower than 18.3 MΩ cm was prepared using a Milli-Q^®^ deionization unit from Merck Millipore (Burlington, VT, USA).

### Instrumentation

Each voltammetric measurement was carried out in a standard voltammetric cell containing 10 mL of 0.1 mol/L aqueous buffer as supporting electrolyte with immersed GCPE, followed by silver/silver chloride reference electrode with 3 mol/L KCl salt bridge from Metrohm (Herisau, Switzerland), and a platinum sheet from Elektrochemické detektory (Turnov, Czech Republic), acting as auxiliary electrode. All mentioned electrodes were connected to a potentiostat/galvanostat (Autolab/PGSTAT101) operated with Nova 1.11 software (Metrohm) (*25*).

### Preparation of different carbon paste electrodes

Four types of carbon pastes were prepared by hand mixing of 0.5 g chemically purified natural graphite powder (particle size of less than 5 µm) from Graphite Týn, (Týn nad Vltavou, Czech Republic), spectroscopic graphite powder (particle size of 10–25 µm) from Ringsdorff-Werke (Bonn, Germany), or glassy carbon powder Sigradur-G (distribution of particle size 5–20 μm) from HTW Hochtemperatur-Werkstoffe GmbH (Maintingen, Germany) and 0.085 g mineral oil from Merck KGaA (Darmstadt, Germany) in ceramic mortar for minimally 20 min. In addition, the carbon paste prepared from natural graphite powder was modified with *w*=5 % multi-walled carbon nanotubes (MWCNTs) from Shenzhen Nanotech Port Co. (Shenzhen, PR China), with a diameter of 10–30 nm, length of 5–15 µm and specific surface area of 40–300 m^2^/g. In addition, glassy carbon paste was enriched with *w*=5 % reduced graphene oxide (RGO), from ACS Material, LLC (Medford, MA, USA), with resistivity ≤0.30 Ω cm and specific surface area 400–1000 m^2^/g.

Each resulting homogenous paste was then pressed into the cavity (diameter of 3 mm) of Teflon^®^ piston-like electrode holder. Herein, it is necessary to note that the height of the paste column must not exceed 2 cm due to difficult extrusion of compact glassy carbon paste. Furthermore, it is recommended not to measure with freshly made paste electrodes due to their rather unstable electrochemical behaviour caused by an incomplete homogenisation, but to leave them under the laboratory conditions for one day. After this self-homogenisation process, all resulting paste electrodes were ready for voltammetric measurements.

### Voltammetric measurements

Repetitive cyclic voltammetric measurements (three cycles) of each 0.5 mmol/L chlorophenol at glassy carbon paste electrode (GCPE) (*26*) in 0.1 mol/L acetate buffer with a pH=4.5, sodium phosphate buffer with a pH=7.0 and ammonium buffer with a pH=10, all with *φ*(EtOH)=5 %, recorded in the potential range from −0.4 to +1.4 V at potential step *E*_step_=2.5 mV and scan rate of *ν*=50 mV/s, were used to investigate electrochemical behaviour of the variously substituted chlorophenols.

Anodic square wave voltammetric measurements of 0.01–10 μmol/L PhOH, 2-CP, 2,6-DCP and 2,4,6-TCP solutions at conventional CPE, GPE, CPE/MWCNTs, GCPE/RGO and GCPE in the tap water, which imitates the brewing water, recorded in the potential range from 0 to +1.0 V at *E*_step_=2.5 mV, potential amplitude *E*_ampl_=25 mV and frequency *f*=4 Hz were used to decide whether the partial contributions of the anodic oxidation of individual chlorophenols are equal to their total content. On the other hand, cathodic square wave voltammetric measurements of wheat beer were carried out at GCPE in the potential range from +1.4 to −0.4 V at deposition potential *E*_dep_=+1.4 V for deposition time *t*_dep_=120 s, equilibrium time *t*_eq_=5 s, *E*_step_=−2.5 mV, *E*_ampl_=−25 mV and *f*=4 Hz.

### Samples

Four different wheat beer samples were chosen for voltammetric analysis, namely Blue Moon (Belgian white beer with 5.4 % alcohol) and Hoegaarden^®^ (4.9 % alcohol), both from Pivovary Staropramen s.r.o. (Prague, Czech Republic), Weizen (4.8 % alcohol) from Primátor a.s., (Náchod, Czech Republic) and Paulaner Weißbier (5.5 % alcohol) from Paulaner Brauerei GmbH & Co. KG. (München, Germany). Tap water was used as a sample of brewing water. Prior to any voltammetric investigation, the beer samples of 50 mL were sonicated for 30 min under laboratory conditions. If the presence of chlorophenols in the samples was not detected electrochemically, they were spiked to have the concentration of 2,6-DCP no more than 100 μmol/L.

### Statistical data processing

Each point of the calibration curve was measured at least five times (*N*=5). The final peak area values were calculated and presented as confidence intervals *x̄*±*st*_1-α_/ where *x̄* is the arithmetic mean, *s* the standard deviation and *t*_1-α_ the critical value of Student's *t*-distribution for ten (4 degrees of freedom) determinations (2.776) at a significance level α of 0.05 (95 % probability). Finally, y-intercept significance of the corresponding calibration curve, plotting the linear dependence of peak area (*A*_p_), presented as average values of five replicate measurements (*N*=5), on actual concentration (*c*) was tested using a statistical software QC-Expert v. 3.3.0.4 (*27*).

## RESULTS AND DISCUSSION

### Electrochemical behaviour of phenol and various substituted chlorophenols

First, phenol had to be electrochemically analysed together with selected chlorophenols using repetitive cyclic voltammetry at glassy carbon paste electrode (GCPE) in several aqueous electrolyte solutions to investigate their anodic oxidation pathways and determine the optimum working conditions for the electrosensing of chlorophenols in the brewing water and top-fermented wheat beer.

**Fig. 1:**
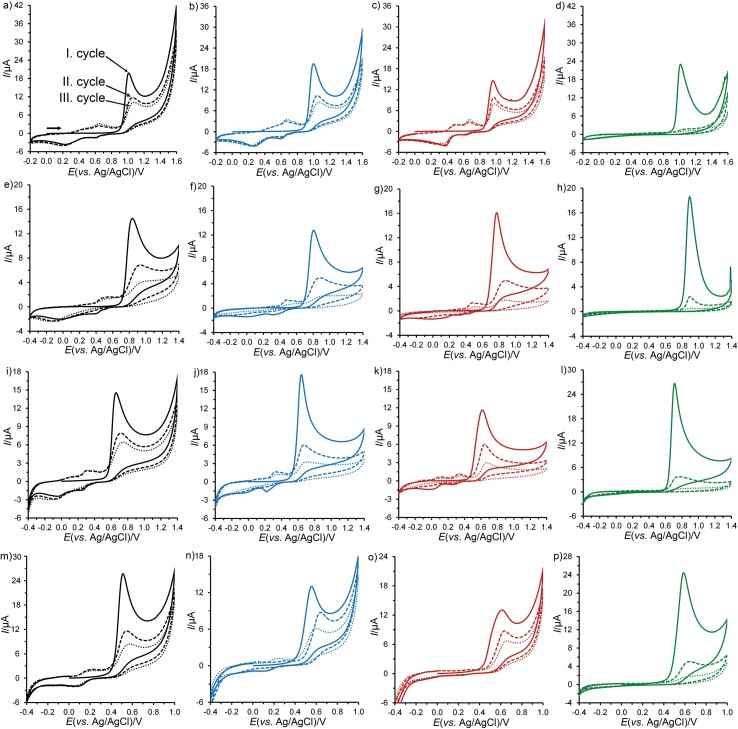
Repetitive cyclic voltammograms (1^st^ cycle: solid line, 2^nd^ cycle dash and 3^rd^ cycle dotted line) of 500 μmol/L PhOH (black), 2-CP (blue), 2,6-DCP (red) and 2,4,6-TCP (green cycles) recorded on GCPE in 0.1 mol/L HNO_3_ (a, b, c and d), AcB with a pH=4.5 (e, f, g and h), SPB with a pH=7 (i, j, k and l) and AmB with a pH=10 (m, n, o and p), all with *φ*(EtOH)=5 % at *E*_step_=2.5 mV and *ν*=50 mV/s. CP, DCP and TCP=chlorophenol, di- and trichlorophenol, respectively, GCPE=glassy carbon paste electrode, AcB=acetate buffer, SPB=sodium phosphate buffer and AmB=ammonium buffer

From the repetitive cyclic voltammograms in Fig. 1, it can be deduced that the electrode reactions of 2-CP, 2,6-DCP and 2,4,6-TCP at carbonaceous electrodes in an aqueous solution are irreversible. As part of the scan rate study for each chlorophenol recorded at 10, 50, 100, 200, 300, 400 and 500 mV/s (*vs.* Ag│AgCl│3 mol/L KCl), it was observed that the anodic peak current response (*I*_p_^a^) increased linearly (R^2^≥0.9952) with the square root of the scan (*ν*). Thus, it can be concluded that electrochemical oxidation reaction is diffusion-controlled. At this point, it is necessary to mention that each cyclic voltammogram was recorded on a newly restored GCPE surface due to subsequent reactions.

Except for 2,4,6-TCP, for which an electrochemical-chemical (EC) reaction mechanism has been proven, for all other chlorophenol reaction mechanism is electrochemical-chemical-electrochemical (ECE), while amounts of following products formed from their anodic oxidations decrease with the increasing number of chlorine atoms on the benzene ring and polarisation rate.

In the first anodic scan (solid cyclic voltammograms in Fig. 1), only one intense oxidation peak can be seen, the peak potential of which shifts to more positive values as the pH of the working medium decreases. This phenomenon is related to two-electron oxidation during which cation intermediates are formed (*28*). These reactive intermediates are subsequently formed by nucleophilic addition of water to form *ortho-* and *para*-quinones, which can be reversibly reduced to the corresponding chlorinated dihydroxyphenols. This fact is evidenced by a pair of redox couples in the subsequent cycles (dashed and dotted cyclic voltammograms in Fig. 1).

**Fig. 2:**
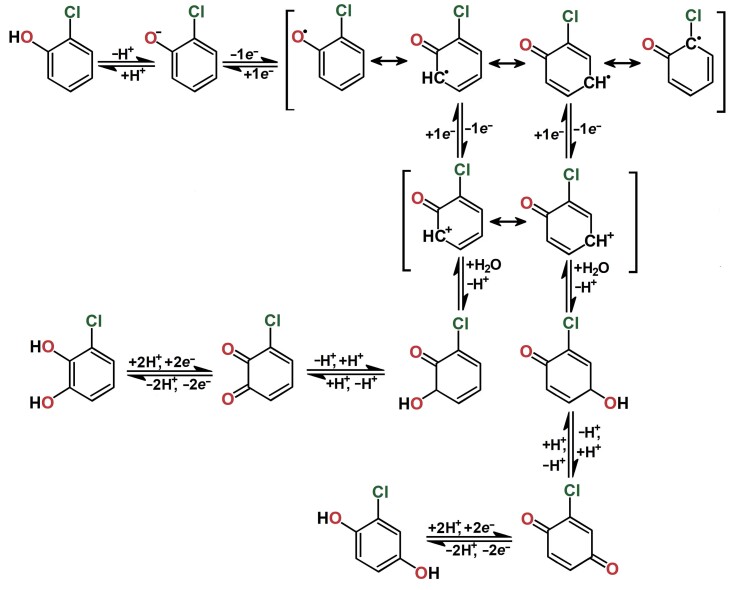
Proposed electrochemical oxidation pathway of 2-chlorophenol

As evident from the proposed anodic oxidation pathway of 2-CP in Fig. 2, the *ortho* and *para* positions on the benzene nucleus must not be occupied to form the above-mentioned chlorinated quinones. However, two well distinguishable redox pairs of 2,6-DCP (see Fig. 1) show that the electrochemical behaviour of chlorophenols is much more complex, although both *ortho* positions are occupied by chlorine atoms. Apparently, chlorine atoms can be replaced by hydroxyl groups when chlorine is released in the form of HCl (*29*) and/or dimers are formed by the coupling of phenoxy radicals (*30*). The formation of such an electrochemically inactive dimer can be considered a product of the anodic oxidation of 2,4,6-TCP because it did not show any subsequent peaks in the second and third cycles. These results indicate that the chlorine atoms are not replaced by hydroxyl groups in the anodic oxidation of 2,4,6-TCP when a 2,4,6-trichlorophenoxy radical is formed, which undergoes a coupling reaction (*31*).

### Possibilities of simultaneous voltammetric determination of chlorophenols

As can be seen from the electrochemical behaviour of the chlorophenols at the GCPE, all investigated chlorophenols show one oxidation peak that can be used for electroanalytical purposes. In general, it can be assumed that the voltammetric analysis should be performed on virgin electrode surface due to the subsequent reactions of their oxidation products. Since a carbon paste electrode fulfils two basic requirements, namely polarisation in the anodic potential window and simple renovation of the electrode surface (*15*), its different analogues and modifications with carbon nanomaterials were examined for the electrochemical detection of chlorophenols in brewing water and several top-fermented wheat beer samples using square wave voltammetry.

**Fig. S1:**
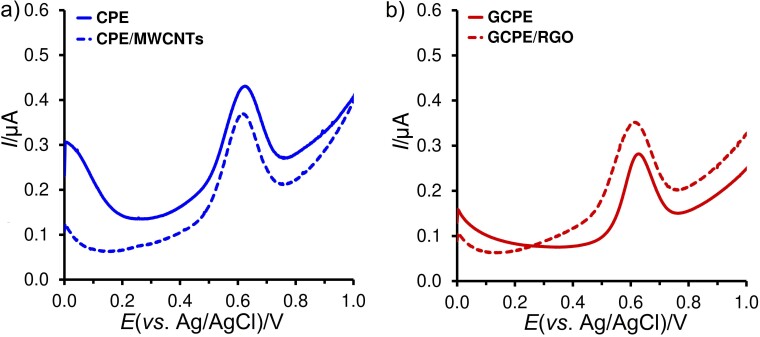
Square wave voltammograms of 0.5 μmol/L 2-chlorophenol obtained in 0.1 mol/L AcB (pH=4) with *φ*(EtOH)=5 % at different carbonaceous composite electrodes in brewing water at *E*_step_=2.5 mV, *E*_ampl_=25 mV and *f*=4 Hz: a) CPE=carbon paste electrode, MWCNTs=multi-walled carbon nanotubes, b) GCPE=glassy carbon paste electrode, RGO=reduced graphene oxide. AcB=acetate buffer

Unfortunately, an admixture of MWCNTs or RGO to CPE or GCPE, respectively, did not lead to the expected narrowing of the anodic peak of 2-CP, as shown in Fig. S1. Another way to achieve the desired separation of the individual chlorophenols in the mixture was to optimise the parameters for square wave voltammetry (SWV). Despite setting very low values for the potential step (2.5 mV) and frequency (4 Hz), which allowed to obtain well-defined shapes of their anodic peaks, it was not possible to distinguish individual chlorophenols in their equimolar mixtures. The values of their anodic peak potentials (*E*_p_^a^) as a function of pH confirm this statement (Table S1).

**Fig. S2:**
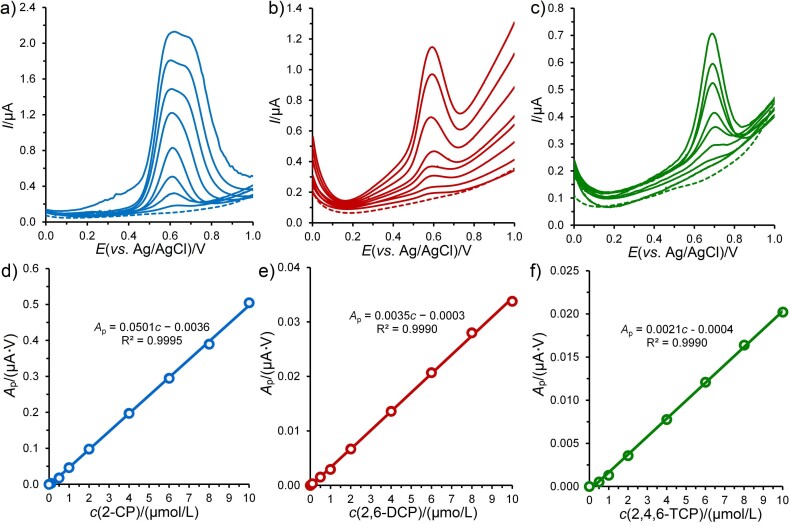
Square wave voltammograms of tap water with 0.1 mol/L AcB (pH=4) and *φ*(EtOH)=5 % spiked with *c*(chlorophenol, CP)/(μmol/L): 0 (dashed line), 0.1, 0.5, 1, 2, 4, 6, 8 and 10 (solid lines), a) 2-CP, b) 2,6-DCP, c) 2,4,6-TCP obtained at GCPE, CPE/MWCNTs and GCPE/RGO, respectively, at *E*_step_=2.5 mV, *E*_ampl_=25 mV and *f*=4 Hz. The corresponding calibration curves (d, e and f) are shown below. GCPE=glassy carbon paste electrode, CPE/MWCNTs=CPE modified with *w*=5 % multi-walled carbon nanotubes, and GCPE/RGO=glassy carbon paste enriched with *w*=5 % reduced graphene oxide. CP, DCP and TCP=chlorophenol, di- and trichlorophenol, respectively

In addition, it was experimentally found that their peak current response depends significantly on the composition of the working solution. As the results of the calibration measurements in Fig. S2 show, the peak current area (*A*_p_) in brewing water (unbuffered working solution) decreases with the number of chlorine atoms on the benzene nucleus because chlorine is an electron-withdrawing group. Thus, the acidic character of chlorophenols increases with a higher number of substituted chlorine atoms.

However, when working in buffered solution with increased ionic strength a relatively comparable sensitivity (~0.045 μA∙V∙L/μmol) for all chlorophenols analysed was achieved, regardless of the type of working electrode used. Since all equations of the linear regression were characterised by a statistically insignificant y-intercept (less than ~0.0005 μA∙V), the standard addition method could be offered for the voltammetric determination of the total amount of chlorophenols, which could be expressed as the concentration equivalent of the most abundant 2,6-DCP.

### Voltammetric analysis of brewing water

Brewing water is considered a crucial component of the beer production because its chemical composition can significantly affect the flavour and quality of the beer. Brewing water usually contains essential minerals (calcium, magnesium, sodium and potassium) and common ions, including bicarbonate, sulfate and chloride. In contrast, chlorine and chloramines can lead to an aftertaste in the beer and must therefore be removed or neutralised before brewing. When tap water is chlorinated, phenols that are formed produce intensely smelling chlorophenols. This is the main reason why chlorophenols can be found in brewing water.

In this study, the use of a slow scan rate (*ν*) of 10 mV/s and a potential amplitude (*E*_ampl_) of 25 mV allowed to sufficiently distinguish the anodic peak of each chlorophenol from the baseline signal of the tap water (dashed square wave voltammograms in Fig. S2), even at a concentration of 0.1 μmol/L. Repeatability, defined as a measure of the ability of the method to produce similar results for multiple preparations of the same sample, was evaluated in a model analysis of brewing water spiked with 1 μmol/L of each chlorophenol. A relative standard deviation (RSD) value of 3.1 % was obtained for five (*N*=5) repetitions.

**Fig. S3:**
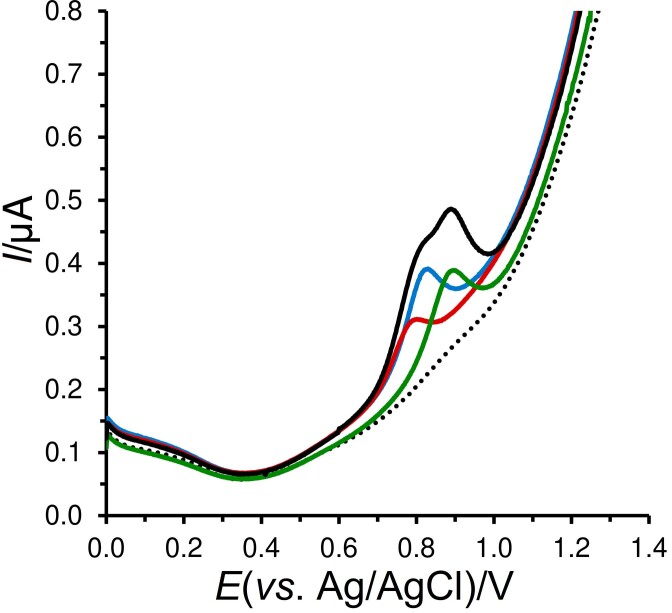
Anodic square wave voltammograms of 0 (black dotted), 1 μmol/L (solid lines) 2-CP (blue) 2,6-DCP (red), 2,4,6-TCP (green) and their equimolar mixture (black curve) obtained at GCPE in 0.1 mol/L acetate buffer (pH=4.5) at *E*_step_=2.5 mV, *E*_ampl_=25 mV and *f*=4 Hz. GCPE=glassy carbon paste electrode, CP, DCP and TCP=chlorophenol, di- and trichlorophenol, respectively

Unfortunately, it was experimentally confirmed that their simultaneous determination in a mixture by direct anodic oxidation is not possible. This fact is illustrated in Fig. S3, where voltammetric recordings for individual chlorophenols and their mixture are shown. It can be concluded that with this approach only the sum of all chlorophenols present in the brewing water can be quantified, which could be expressed as the concentration equivalent of 2,6-DCP.

### Voltammetric analysis of wheat beer

In general, insufficiently removed chlorine can produce undesirable chlorophenols during the fermentation phase of the brew. Because beer usually contains high amounts of naturally occurring phenolic substances, the use of direct anodic SWV as in brewing water is not an option. For this reason, the cathodic reduction of anodically oxidised chlorophenols was tested at least as a possibility for their selective voltammetric detection in the complex matrix of wheat beer. During the potentiostatic oxidation of 2,6-DCP, the working solution was stirred to increase the amount of accumulated oxidation product. Since the pH of beer in practice is usually in the range of 4.3-4.7, the wheat beer samples enriched with 1 μmol/L 2,6-DCP were diluted at different ratios with 0.1 mol/L acetate buffer (pH=4.5).

**Fig. 3:**
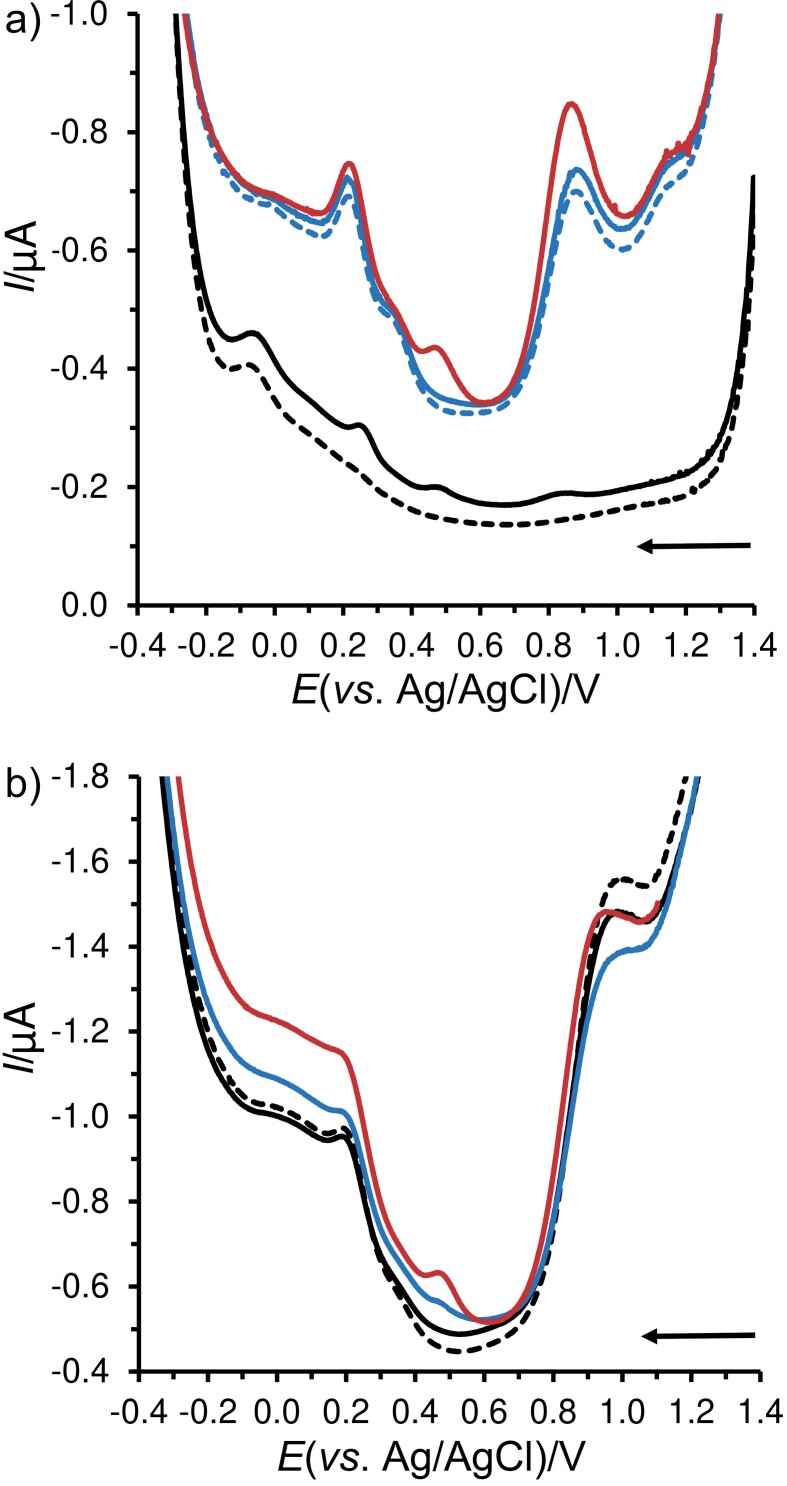
Square wave voltammograms of: a) 0.1 mol/L AcB (pH=4) without (dashed black line) or with 1 μmol/L 2,6-DCP (solid black line), tenfold diluted Weizen without (dashed blue line) or spiked with 1 (solid blue line) and 10 μmol/L 2,6-DCP and b) Paulaner Weißbier without (dashed black line) or spiked with 10 (solid black line), 50 (solid blue line) and 100 μmol/L 2,6-DCP (solid red line), all recorded on GCPE at *E*_dep_=+1.4 V, *t*_dep_=120 s, *t*_eq_=5 s, *E*_step_=−2.5 mV, *E*_ampl_=−25 mV and *f*=4 Hz. GCPE=glassy carbon paste electrode. CP, DCP and TCP=chlorophenol, di- and trichlorophenol, respectively

Fig. 3a shows that 2,6-DCP can generate up to three cathodic peaks at +0.841, +0.487 and +0.272 V when previously oxidised potentiostatically at +1.4 V for 120 s. Surprisingly, the cathodic peak at +0.487 V was not observed in all beer samples, whether diluted (blue dashed voltammogram in Fig. 3a) or not (black dashed voltammogram Fig. 3b). The magnitude of this cathodic signal depended on the concentration of 2,6-DCP, while it was not possible to detect concentrations lower than 50 μmol/L 2,6-DCP in the undiluted beer samples.

**Fig. S4:**
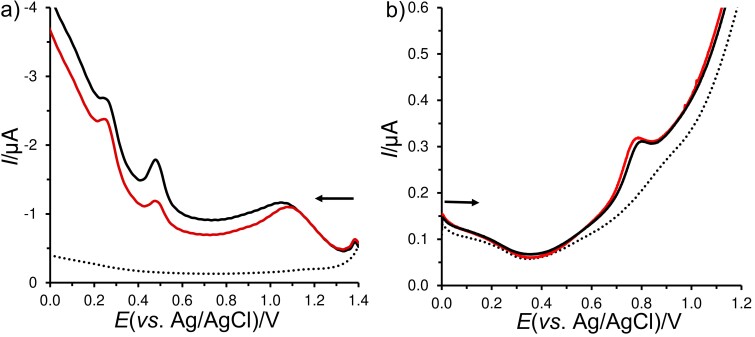
Cathodic (a) and anodic (b) square wave voltammograms of 0 (dotted black) and 1 μmol/L 2,6-DCP recorded on GCPE in 0.1 mol/L acetate buffer (pH=4.5) without (solid black) and with (red curve) *φ*(EtOH)=5 % at *E*_dep_=+1.4 V, *t*_dep_=120 s, *t*_eq_=5 s, *E*_step_=−5 mV, *E*_ampl_=−25 mV, and *f*=60 Hz. GCPE=glassy carbon paste electrode, DCP=dichlorophenol

Fortunately, it was possible to detect and quantify even 1 μmol/L 2,6-DCP in the model sample through further investigations and partial optimisation of the electrochemical detection, in particular by adjusting the SWV operating parameters. It was also found that *φ*(EtOH)=5 % has a statistically significant effect on the height of the reduction peak in cathodic stripping voltammetry (Fig. S4a), in contrast to direct anodic voltammetry, where this effect is negligible (Fig. S4b). This phenomenon can be attributed to the higher solubility of 2,6-DCP in aqueous-ethanol mixtures, and thus to its lower tendency to accumulate on the surface of GCPE by polar/nonpolar interactions, which are also affected by electrostatic interactions when a positive deposition potential (*E*_dep_) is applied. In any case, this electrochemical study suggests that a stripping voltammetric method for the selective determination of 2,6-DCP in beer could be developed in the near future.

## CONCLUSIONS

The present electrochemical study confirmed the impossibility of the simultaneous voltammetric determination of phenol with chlorophenols and at the same time showed the hidden flaws of the previously developed electroanalytical methods based on their anodic oxidation. It has been experimentally proven that only the total amount of chlorophenols can be determined using anodic square wave voltammetry (SWV) in soft drinking water, which is commonly used as brewing water. Nevertheless, it was possible to distinguish the artificial addition of 2,6-DCP even in the complex matrix of wheat beer using a cathodic SWV with potentiostatically controlled accumulation. This breakthrough finding could serve as a basis for the development of selective and sensitive voltammetric methods suitable for such samples.

## Appendix

**Table S1:** Overview of anodic peak potentials (*E*_p_^a^) of phenol and chlorophenols depending on the pH of different working solutions

Supporting electrolyte	pH	*E*_p_^a^/V						
		PhOH		2-CP	2,6-DCP		2,4,6-TCP	
HNO_3_	1.0	1.017	1.007			0.972		1.017
AcB	4.5	0.851	0.811			0.775		0.896
SPB	7.0	0.670	0.645			0.629		0.715
AmB	10.0	0.514	0.540			0.599		0.594

*c*(supporting electrolyte)=0.1 mol/L. AcB=acetate buffer, AmB=ammonia buffer and SPB=sodium phosphate buffer. CP, DCP and TCP=chlorophenol, di- and trichlorophenol, respectively
